# The Halophyte Dehydrin Sequence Landscape

**DOI:** 10.3390/biom12020330

**Published:** 2022-02-19

**Authors:** Siwar Ghanmi, Steffen P. Graether, Moez Hanin

**Affiliations:** 1 Plant Physiology & Functional Genomics Research Unit, Institute of Biotechnology, University of Sfax, Sfax 3038, Tunisia; ghanmi.siwar11@gmail.com; 2 Department of Molecular and Cellular Biology, University of Guelph, Guelph, ON N1G 2W1, Canada; 3 Graduate Program in Bioinformatics, University of Guelph, Guelph, ON N1G 2W1, Canada

**Keywords:** halophytic plants, dehydrin, in silico analysis, abiotic stress, salt stress

## Abstract

Dehydrins (DHNs) belong to the LEA (late embryogenesis abundant) family group II, that comprise four conserved motifs (the Y-, S-, F-, and K-segments) and are known to play a multifunctional role in plant stress tolerance. Based on the presence and order of these segments, dehydrins are divided into six subclasses: YnSKn, FnSKn, YnKn, SKn, Kn, and KnS. DHNs are rarely studied in halophytes, and their contribution to the mechanisms developed by these plants to survive in extreme conditions remains unknown. In this work, we carried out multiple genomic analyses of the conservation of halophytic DHN sequences to discover new segments, and examine their architectures, while comparing them with their orthologs in glycophytic plants. We performed an in silico analysis on 86 DHN sequences from 10 halophytic genomes. The phylogenetic tree showed that there are different distributions of the architectures among the different species, and that FSKn is the only architecture present in every plant studied. It was found that K-, F-, Y-, and S-segments are highly conserved in halophytes and glycophytes with a few modifications, mainly involving charged amino acids. Finally, expression data collected for three halophytic species (*Puccinillia tenuiflora*, *Eutrema salsugenium*, and *Hordeum marinum*) revealed that many DHNs are upregulated by salt stress, and the intensity of this upregulation depends on the DHN architecture.

## 1. Introduction

Plants can be exposed to a wide range of abiotic stresses, such as drought, salinity, high temperature, and cold [1]. Salt stress is one of the major environmental constraints on plant growth, where about 6% of agricultural lands are affected by salinity [2]. The harmful effects of salinity are generally associated with the low osmotic potential of the soil solution and the high level of sodium toxicity, which causes multiple negative effects on the metabolism, growth, and development of plants at molecular, biochemical, and physiological levels [3]. As sessile organisms, plants have developed several mechanisms in response to these unfavorable conditions during their growth. One example is osmotic adjustment by accumulating various osmoprotectants (e.g., proline, glycine betaine, soluble sugars) to limit water loss, and hence maintain cell turgor, along with preserving protein structure and membrane integrity [4,5]. To survive under salt stress conditions, some plants adopt the “exclusion strategy”, by excluding sodium from the cytoplasm and/or by sequestering it to the vacuolar compartment [2].

Faced with salt stress, not all plant species respond equally, and therefore have different tolerance levels. Plants can be divided into salt-sensitive glycophytes or salt-tolerant halophytes. Halophytes are defined as all plant species with the ability to complete their life cycles in soil containing at least 200 mM NaCl [6]; they are estimated to represent at most 2% of terrestrial plant species [7]. Several studies have revealed how plants function by comparing different species. The strong salt tolerance of the *Brassicaceae Thellungiella halophila* (salt cress) in comparison with the allied species *Arabidopsis thaliana* (thale cress) was attributed to a tight control of Na^+^ and K^+^ uptake and higher levels of the compatible osmolyte proline. Injury of young photosynthetic leaves and acceleration of their senescence can be also caused by soil salinization, as the Na^+^ cation is toxic when it accumulates in cell cytosol, resulting in ionic imbalance and metabolic toxicity in transpiring leaves [8]. In addition, a high number of genes is differentially expressed, which includes late embryogenesis abundant (LEAs) genes, which are highly activated in *Thellungiella halophila* [9,10].

Late embryogenesis abundant proteins were initially discovered in cotton seeds, and were found to accumulate during the later stage of seeds development, which gave them the ability to tolerate abiotic stress [11,12]. Since then, LEAs have been found in all examined plants (vascular and non-vascular) and are also distributed in a wide range of organisms (algae, yeast, cyanobacteria, and brine shrimp) [13]. In mature embryos, as well as in stressed cereal seeds, they can represent up to 1% of the total soluble proteins [12,14]. Dehydrins (DHNs) form the best-characterized LEAs subfamily (known also as group II LEAs) [15,16]. DHNs have highly hydrophilic sequences, with a high content of Gly, Ala, and Ser, but lack Cys and Trp, and contain few hydrophobic amino acids [17,18]. Structural analysis by circular dichroism showed that they are intrinsically disordered proteins (IDPs) [13,19,20,21], and therefore have little secondary structure and almost no tertiary structure [19]. These proteins are characterized by a wide range of molecular masses, ranging from 9 to 200 kDa [22].

Dehydrins are defined by the presence of a conserved segment called the K-segment. Rich in Lys, the motif can be defined by the sequence [XKXGXX(D/E)KIK(D/E)KXPG], with the most conserved residues being in the middle of this segment (Lys-Ile-Lys-Glu). The motif is able to interact with membranes and other biomacromolecules to protect them from stress damage [23,24]. The F-segment is a new motif discovered by [25], characterized by the presence of a pair of hydrophobic Phe residues at the core of the sequence. Dehydrins have two additional conserved motifs, the Y-segment, [D(D/E)(Y/H/F)GNP] near the N-terminus and the S-segment ([LHR(S/T)GS_4–6_(S/D/E)(D/E)_3_]). The Y-segment is known by the presence of a Tyr amino acid in the core of this motif; although, other aromatic amino acids, such as His and Phe, are also found at this position. This motif has sequence similarity to the nucleotides binding site in plant chaperones [26], but direct testing of this showed that the motif does not bind nucleotides [27]. Its function is therefore still unknown. For the S-segment, its phosphorylation can influence nuclear localization [15,26,28]. Dehydrins also contain less conserved ϕ-segments, which are enriched with polar amino acids, Gly, or a combination of Pro and Ala, and are located between the conserved segments [29]. Depending on the presence, absence, and number of repetitions of these segments, DHNs can be classified into six distinct groups: Kn, SKn, KnS, YnSKn, FSKn, and YnKn (with n being the number of that segment) [30,31].

Dehydrins play relevant role in enhancing abiotic stress tolerance [15,32]. This beneficial effect can be attributed to the conserved segments of DHNs that seem to have a large number of protective functions in plants, including enzyme protection from damage caused by environmental stress [33,34,35,36]. Several studies have suggested that the K-segment contributes to this cryoprotective function [30,37,38]. Moreover, these peptides perform a chaperone activity able to protect proteins from denaturing or forming inactive aggregates during stress [39].

In addition to enzyme protection, other in vitro studies highlight multiple roles for dehydrins, including the protection of membranes and DNA [27,30,40,41], where the K-segment plays an important role. With regard to membranes, several studies have also shown that the K-segments in dehydrin gain α-helical structure in the presence of membranes [42,43,44], and that they are able to protect membranes from abiotic stresses [45,46,47].

Despite their relevant structural and functional properties, that are tightly associated with various stress tolerance mechanisms in many plants, DHNs are rarely studied in halophytes so that their contribution in salt stress adaptation remain largely unknown. In this work, we performed the first genomic study on halophytic DHN sequences to discover new segments, and to examine their architectures in comparison with their orthologs in glycophytic plants. Our in silico analyses showed that FSKn is the only architecture present in all the ten halophytes studied here. In addition, we leveraged expression data collected from three halophytic species (*Puccinillia tenuiflora, Eutrema salsugenium,* and *Hordeum marinum*) to point out that many DHNs are upregulated by salt stress, and the intensity of this upregulation depends on the DHN architecture. The current study on the halophytic DHNs will guide our future research in understanding the mechanism by which salt-tolerant plants are able to withstand this abiotic stress.

## 2. Materials and Methods

The current work aims to perform a comprehensive bioinformatics study on DHNs found in several different halophytic species (*Eutrema salsugenium*, *Cakille maritima*, *Chenopodium quinoa*, *Hordeum vulgare*, *Daucus carota*, *Zostera marina*, *Phoenix dactylifera*, *Carex littledalei*, *Asparagus officinalis,* and *Lactuca saligna*). The approach used is similar in part to that described in [17,48]. In brief, assembled genome sequences were collected from the Phytozome and NCBI genome browser. The K-segment identified in [48] was used as the query sequence for FIMO with a threshold value of 10^−7^ to assemble a set of halophytic DHNs. All other options were left at their default settings. The resulting matches were run through MEME (any number of motifs with a width of 15 residues) to create a tentative halophytic K-segment. FIMO was re-run with the new K-segment and a stricter threshold of 10^−8^. Individual sequences that contained K-segment motifs with a *p* > 6 × 10^−11^, low-complexity sequences and proteins rich in Glu, Lys, and Leu were excluded from the dataset.

Because KnS dehydrins contain K-segments that can be different from those of other architectures, an additional search for potential DHN sequences was performed by using the KnS dehydrins from [48], as the query search sequence in a BLAST search of all of the halophytic genomes. The resulting matches with an *E*-value ≤ 0.01 were added to the DHN sequence database.

To create LOGO motifs of the K-, F-, Y-, and S-segments, MEME was run on the sequence database. Different widths were chosen for the K- (15 residues), F- (14 residues), and Y-segments (8 residues). For the S-segment, the width was allowed to vary between 7 and 15 residues. For all MEME searches, the “any number of repeats” mode was used, with a search limit of 10 motifs. All other parameters were left at their default values.

The architectures were determined using these halophytic motifs in a MAST search. A species tree was inferred using the PhyloT server (https://phylot.biobyte.de (accessed on 21 October 2021)). The phylogenetic tree was generated using the NCBI taxonomy database. Analysis of the physiochemical properties was performed as described in [17]. In brief, the pI, GRAVY value, and Mr were obtained from the Geneinfinity web server (http://www.geneinfinity.org/sms/sms_proteiniep.html (accessed on 21 October 2021)). The FoldIndex was obtained from the Proteopedia web server (https://fold.proteopedia.org (accessed on 21 October 2021)). The property values were visualized using the “bean plot” package in R [49].

Expression data were collected from the previous reports [50,51,52]. Using gene IDs available in these studies, we have identified the protein sequences of dehydrins in the Phytozome v13 database and determined their architectures by using MEME.

## 3. Results and Discussion

### 3.1. Dehydrin Architectures

**Figure 1:**
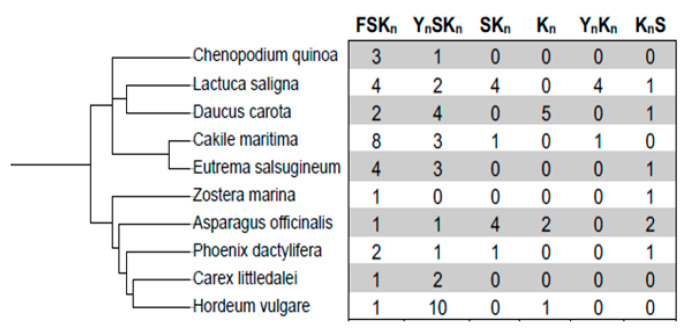
Phylogenetic tree of halophytic species used in this study. The left side of the figure shows a rooted phylogenetic tree of the halophytes, while the right side shows a count of the different architectures in each of the individual species.

We examined the distribution and the number of architectures of DHNs (FSKn, YnSKn, SKn, Kn, YnKn, KnS) within the 10 available halophytic-plant-assembled genomes. To perform this analysis, we have inferred a phylogenetic tree and an accompanying table to indicate the distribution and number of DHN architectures within each of the species (Figure 1). From the table, we can see that there are different distributions of the architectures among the different species. FSKn is the only architecture present in each of the examined species, with the number of copies varying from 1 to 8. Moreover, FSKn and YnSKn are the two most common architectures as they were found in 10 and 9 species, respectively. Other architectures, especially YnKn and Kn (i.e., those lacking S-segments), are weakly represented in our halophytic populations. 

In glycophytes, more variable distribution of the dehydrin architectures was observed depending on species, as previously reported by Malik et al., 2017. For example, in grasses, only YnSKn, SKn, and KnS architectures are found, while in *Brassicacea*, all five major architectures may be present (Kn, YnSKn, YnKn, and SKn).

Whether specific architectures are associated with higher stress tolerance in halophytes is a difficult question to answer, since it has been challenging to perform the same in glycophytes. However, some insights can be provided from this analysis. For instance, in the 2 species, *Cakille maritima* and *Eutrema salsugenium,* that can survive at 500 and 700 mM NaCl, respectively [53,54], the FSKn architecture is quite numerous, with 8 and 4 copies, respectively, whereas in *Hordeum vulgare,* which is less tolerant to salt stress (200 mM NaCl) [55] (https://www.sussex.ac.uk/affiliates/halophytes/ (accessed on 15 November 2021)), only one FSKn DHN, but many YnSKn DHNs were found. The absence of YnSKn DHN in *Zostera marina* is interesting, and may reflect the fact that it grows in seawater rather than on land, suggesting that this species may have evolved different mechanisms to respond to salt stress. In terms of environments, it is also interesting to note which species have the Kn architecture. It has been shown that Kn expression is correlated with cold tolerance [56]. The presence of this architecture only in *Daucus carota, Asparagus officinalis*, and *Hordeum vulgare* may therefore reflect their cold hardiness rather than their salt tolerance.

### 3.2. Comparative Analysis of the Conserved DHN Motifs in Halophytes and Glycophytes

**Figure 2:**
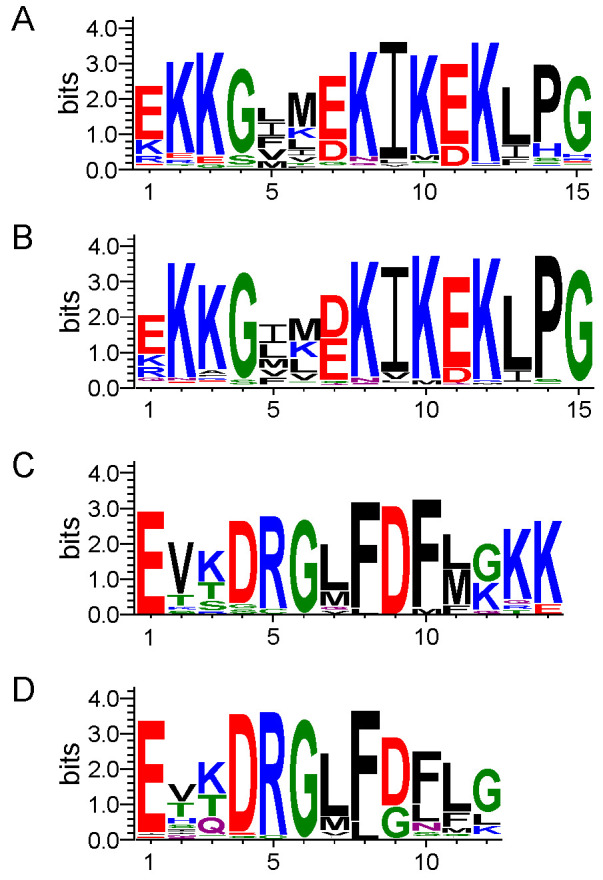
K- and F-segments from halophytic and glycophytic plants. LOGO representations of conserved dehydrin segments. Amino acids are grouped by color according to their physiochemical property. Blue—positively charged (Lys, Arg, His); red—negatively charged (Asp, Glu); black—hydrophobic (Ala, Val, Leu, Ile, Pro, Phe, Met); green—polar (Gly, Ser, Thr, Tyr, Cys); purple—neutral (Asn, Gln). The height of the amino acids corresponds to their conservation at that position. (**A**) K-segment from halophytes. (**B**) K-segment from glycophytes. (**C**) F-segment from halophytes. (**D**) F-segment from glycophytes.

We next used the dehydrin sequence dataset as input for the MEME program to search for the K-, F-, S-, and Y-segments, and to compare DHN motifs between halophytes and glycophytes. The LOGO representations of the K- and F-segments are shown in Figure 2A,C, with the amino acid frequencies indicated in Table S1 and Table S2, respectively. As noted in the Introduction, DHNs contain the K-segment, rich in Lys, and our results confirm the presence of a K-segment in all 86 dehydrin sequences. For comparison, the K-segment of non-glycophytic plants is shown in Figure 2B. For the most part, the two motifs are similar, with some minor differences. The most obvious is in position 7, where a Glu is preferred in halophytic plants rather than Asp. There also appears to be more Glu residues in positions 2 and 3, whereas glycophytes show Lys more frequently. Lastly, there are a small number of Phe at position 13. This is unusual, because generally Phe is an aromatic residue that promotes structuring, and is quite rare in dehydrins outside of the F-segment.

An analysis of the F-segment from halophyte species (Figure 2C) was compared with those from glycophytes (Figure 2D). The LOGO reveals the presence their short, palindromic sequence—GLFDFLG—with less conserved positions flanking both ends of this central core. In the halophytic F-segment, there is an extension of the conserved motifs by two Lys residues at the C-terminus (Figure 2C). Additional significant differences were detected as well; halophytic DHNs have at position 12 more Lys, while at position 9, Asp dominates while there is a mix of Asp and Gly in glycophytes. Lastly, Ser seems to have replaced Gln in position 3 in terms of being the third most frequent amino acid.

The LOGO representation in Figure 2A,C of the K- and F-segments reveals that the sequence of this motif is similar to that outlined in the literature for glycophytes. This includes conservation in the K- and F-segments of their hydrophobic character, which plays important roles in the cryoprotective activities of dehydrins. Previous reports focused on the truncated forms of the F- and K-segments have shown their involvement in enzyme protection [35,41,57,58,59]. This is not surprising, since these mechanisms are likely similar between halophytes and glycophytes, such that the sequences must be fairly conserved.

While no major shifts were observed in sequence conservation, most of the observed changes in halophytic K- and F-segments revolve around charged residues (both acidic and basic ones). The interaction of DHNs with membranes has been shown to be sensitive to the salt concentration [45]. The presence of the two Lys residues in the F-segment may therefore be important for membrane binding in the presence of higher salt concentrations. Similarly, the acidic charges may be important for cryoprotection. An increase in the number of acidic residues has also been observed in halophilic bacteria, where it has been suggested that their presence is important for water binding during salt stress [60].

**Figure 3:**
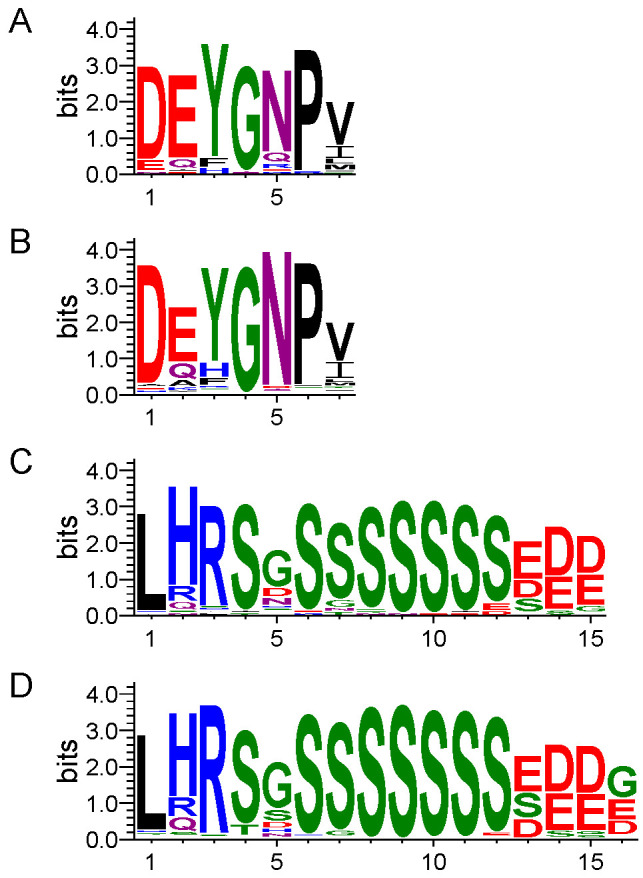
Y- and S-segments from halophytic and glycophytic plants. LOGO representations of conserved dehydrin segments. Amino acids are grouped by color according to their physiochemical property. Blue—positively charged (Lys, Arg, His); red—negatively charged (Asp, Glu); black—hydrophobic (Ala, Val, Leu, Ile, Pro, Phe, Met); green—polar (Gly, Ser, Thr, Tyr, Cys); purple—neutral (Asn, Gln). The height of the amino acids corresponds to their conservation at that position. (**A**) Y-segment from halophytes. (**B**) Y-segment from glycophytes. (**C**) S-segment from halophytes. (**D**) S-segment from glycophytes.

We next examined the Y- and S-segment motifs, and again compared them between halophytes and glycophytes. As shown in Figure 3A, the LOGO representation of the MEME output of the Y-segment reveals that Tyr was a very frequently found amino acid at position 3 in halophytes, even more so than in glycophytes (Figure 3B). Nevertheless, as an alternate, His and Phe residues were still found at this position, with the Phe occurring more often. The presence of these three amino acids suggests that it is the aromatic character that is important at this position; although, interestingly, Trp has never been detected [17]. Among the three amino acids, only Trp has an indole ring on its side chain while the two others are single-ring aromatics. The hydrophobic large indole ring of Trp is known to play a crucial role in protein folding, a feature that His or Phe fail to perform as reported through Trp-cage-folding modeling studies [61]. Moreover, conservation of the three amino acids Asp, Gly, and Asn, at positions 1, 4, and 5, respectively, is also maintained, with Asp and Glu at positions 1 and 2 being even more common in halophytes. The other difference appears to be minor; at position 5, Asn is less conserved, with Gln and Arg occurring more often than in glycophytes. The amino acid frequencies for the halophytic Y-segment are shown in Table S3.

The S-segments found in halophytes and glycophytes share very conserved sequences. In Figure 3C,D, we see the halophytic and glycophytic motifs; the S-segments are 15 residues in length, while they are 16 residues for glycophytes, and have an additional Asp Glu or Gly at the C-terminal end. The S-segments have a variable number of Ser residues, which is challenging to show in LOGO representation; nonetheless, it appears that halophytic S-segments are probably one residue shorter. In addition, there seems to be slightly better conservation of the His and Arg in position 2 and 3 in glycophytes. For the halophytic S-segment, the amino acid frequencies are shown in Table S4. Compared with the increased number of charged residues in the F- and K-segments, there is no clear pattern that could be detected in the Y- and S-segments. In this case, it is likely that the function of these motifs does not need to be drastically altered for halophytes.

### 3.3. Physiochemical Characteristics of Dehydrins

Previous studies have shown that the overall biochemical properties of DHN sequences depend largely on their architecture [17]. While the motifs themselves are mostly conserved between the different glycophytic architectures, the intervening ϕ-segments make the most contribution to the overall biochemical properties. Therefore, we address the questions, here, of how these properties of halophytic DHNs can be affected by the architecture, and how these may differ from glycophytes. In this study, four biochemical properties were analyzed and compared: molecular mass (a measure of their size), isoelectric point (a measure of their charge), GRAVY score (a measure of their net hydrophobicity), and FoldIndex (a measure of their propensity to fold).

**Figure 4:**
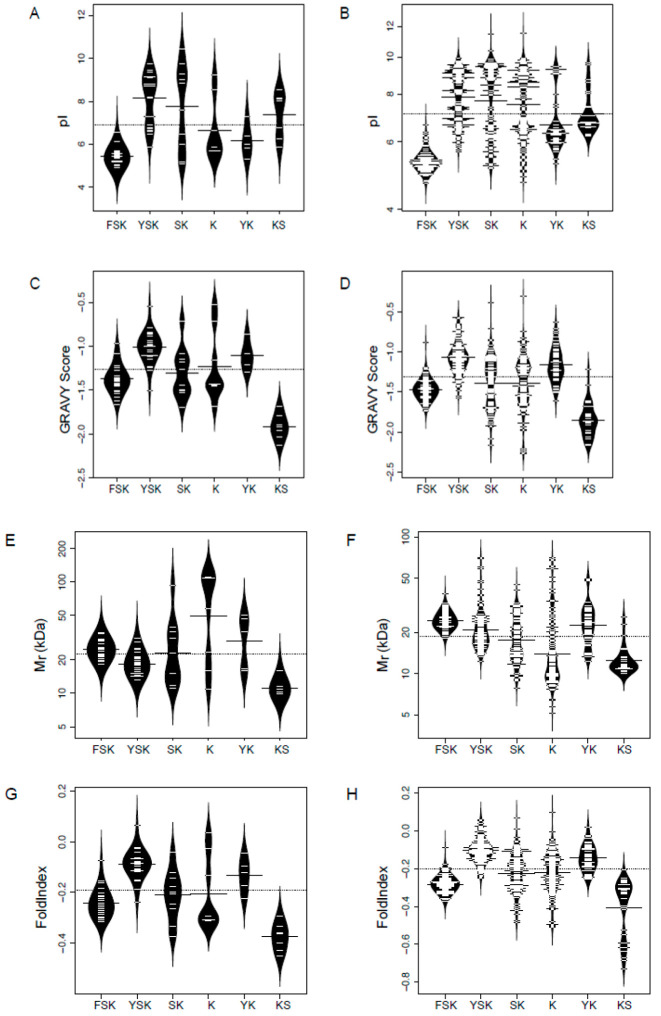
Physiochemical properties of halophytic dehydrins for each architecture. (**A**,**B**) Isoelectric point (pI). (**C**,**D**) GRAVY score. (**E**,**F**) FoldIndex score. (**G**,**H**) Molecular weight (M_r_). Halophytic proteins are shown on the left while glycophytic proteins are shown on the right. The thin bars show the value of an individual protein, the wider black bar shows the mean value of an architecture, and the dotted line shows the mean value of all protein sequences. The violin shape shows the density of the values. The y-axis scale for M_r_ is logarithmic, while all other y-axes are linear.

First comparing within halophytic dehydrins, we see that the distribution of pI scores (Figure 4A) is bimodal for four architectures (YnSKn, Kn, SKn, and KnS), where YnSKn and SKn show a basic pI value centered around pH 8, while Kn has a pI around 6.5 and KnS has a pI around 7.5. The pI distribution of FSKn and YnKn are unimodal with an acidic pI around 5 and 6, respectively. A comparison with glycophytes (Figure 4B) shows that most of the architectures have similar distribution of pI values. The two exceptions are the Kn and KnS architectures. Kn appears to be more acidic in halophytes, while KnS appears to be more basic. As with charged residues in the F- and K-segments, these changes in pI may be necessary to compensate for the high-salt environment.

GRAVY analyses of halophytes and glycophytes (Figure 4C,D) indicate that all DHNs have negative GRAVY scores, which are typical for hydrophilic proteins. The K-architecture shows a slight shift in distribution towards both less and more negative scores in halophytes, but all other architectures show a very similar range and distribution between the two; KnS shows a unimodal distribution, with an average near −2.0. The remaining architectures have very similar distribution shapes and averages, likely reflecting the need for the DHNs to retain their same overall hydrophilic character, no matter the abiotic stress type.

We also calculated the FoldIndex values for these DHNs, which investigate the mean net charge and hydrophobicity of a given protein sequence to predict if it is likely to fold [62]. Figure 4E,F show that the FoldIndex scores largely depends on the GRAVY score, with negative scores predicting that dehydrins are unlikely to fold, and hence be intrinsically disordered. An exception to this is the KnS architecture, where glycophytes had a few proteins that appear to be more disordered than halophytic ones.

The Mr plots (Figure 4G,H) showed that FSKn, YnSKn, and KnS have a unimodal distribution, having an average of about 20, 18, and 10 kDa, respectively. The Kn and YnKn distribution are bimodal, with the Mr centered at 100 and 50 kDa, respectively. Comparative analysis between halophytes and glycophytes show that the Mr of the YnSKn, YnKn, and KnS look more or less the same, while SKn appears to be slightly smaller and Kn appears to be slightly larger in halophytes. We have previously found that larger DHNs are better at protecting enzymes from cold damage [63], but the impact on protection from salt damage is not yet known.

### 3.4. Dehydrin Expression Profiling

**Figure 5:**
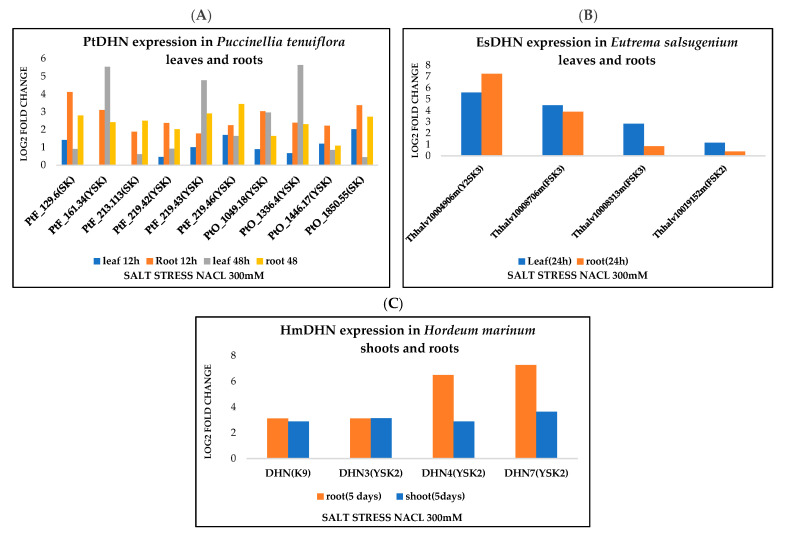
Dehydrin gene expression under salt stress. (**A**) Relative gene expression of several DHN architectures after 12 h and 48 h of salt stress (Nacl 300Mm) in *Puccinellia tenuiflora* leaves and roots [50]. (**B**) Relative gene expression of DHN architectures in *Eutrema salsugenium* leaves and root after 24 h of salt stress (NaCl 300 mM) [51]. (**C**) Relative gene expression of DHN architectures in *Hordeum marinum* shoots and roots after 5 days of salt stress (NaCl 300 mM) [52].

We addressed here the changes in expression levels of various halophytic DHN architectures, under salt stress. Expression data available for halophyte plants are scarce but we have managed to collect expression data available in the literature and NCBI databases. This information revealed that DHNs are upregulated under various abiotic stresses, such as salt stress, in different tissues of halophytic plants. Interestingly, it seems that for some DHNs, there is preference for some architectures by stress. We investigated, for instance, the changes in expression under salt stress of different architectures in the leaves, shoots, and roots of three species (*Puccinellia tenuiflora Eutrema salsugenium,* and *Hordeum marinum*), according to the previously reported studies [50,51,52]. As shown in Figure 5, YnSKn dehydrins are significantly more induced by salt stress than SKn in *Puccinellia tenuiflora* in leaves and roots with log2-fold changes reaching up to 5.63 and 3.44, respectively (Figure 5A). In the case of *Eutrema salsugenium*, we focused here on the expression of three dehydrins, FSK2, FSK3, and Y2SK3, under salt stress. The results revealed that the expression patterns of these three DHNs look different. Y2SK3 and FSK3 were strongly induced, with log2-fold induction reaching up to 5.59 and 4.46, respectively, in leaves. Y2SK3 was even more induced in roots (log2-fold change up to 7.2). On the other hand, FSK3 and FSK2 were more expressed in the leaves than in the roots (Figure 5B), with the first being more salt induced (3.8 versus 0.3).

The comparison between the expression levels of two architectures in the shoots and roots of *Hordeum marinum* (K9 and YSK2) revealed that YSK2 dehydrins (at least DHN4 and DHN7) seem to be more induced by salt stress than K9 (Figure 5C). Although preliminary, these results show differential DHN gene expression between different architectures under salt stress conditions, and, even within the same architecture, the expression of one DHN can vary substantially (Figure 5). These results are broadly similar to what was shown previously [17,48]. However, it seems that the two architectures (FSKn and YSKn), that are the most salt-stress-induced in *Puccinellia tenuiflora Eutrema salsugenium,* and *Hordeum marinum*, are also the most frequent ones in halophytes, as indicated in Figure 1. Such finding suggests that both YSKn and FSKn might be more requested under salt stress conditions.

## 4. Conclusions

Our extensive study of halophyte and glycophyte plant dehydrins showed that their structures are highly conserved. Almost all dehydrin architectures (Kn, SKn, YnKn, SKn, KnS, and FSKn) are present in halophytic and glycophytic plants. Analysis by MEME program revealed that lysine residues are highly conserved in the K and F segments, with an extension of two Lys residues at the C-terminus of the halophytic F-segment and the increased conservation of basic and charged residues. The Y- and S-segments also share very conserved sequences between glycophytes and halophytes. However, and in contrast to glycophytes, we found that the dehydrin architectures are not randomly distributed among halophytes, with the FSKn and YnSKn being the two most common architectures. Interestingly, we found that these two particular architectures seem to be more associated with salt stress tolerance in halophytes, since their expression exhibited the highest level of induction during salt stress. These results give important guidelines in better understanding the mechanism by which halophytic plants are able to tolerate salt.

## Supplementary Materials

The following are available online at https://www.mdpi.com/article/10.3390/biom12020330/s1. Table S1: Amino acid frequencies of the halophytic K-segment; Table S2: Amino acid frequencies of the halophytic F-segment; Table S3: Amino acid frequencies of the halophytic Y-segment; Table S4: Amino acid frequencies of the halophytic S-segment.
