# Supplementary material for: The Halophyte Dehydrin Sequence Landscape
Source: Biomolecules. 2022 Feb 19;12(2):330. doi: 10.3390/biom12020330 (PMC8869203; doi:10.3390/biom12020330)
Supplement: Supplementary file 1 [file biomolecules-12-00330-s001.zip › biomolecules-1577451-supplementary.pdf]

## Supplementary Tables

### Table S1. Amino acid frequencies of the halophytic K-segment.

| | Amino Acid | | | | | | | | | | | | | | | | | | | |
|---|---|---|---|---|---|---|---|---|---|---|---|---|---|---|---|---|---|---|---|---|
| Residue Position | A | C | D | E | F | G | H | I | K | L | M | N | P | Q | R | S | T | V | W | Y |
| 1 | 0.02 | 0.00 | 0.03 | 0.64 | 0.00 | 0.00 | 0.01 | 0.00 | 0.19 | 0.00 | 0.00 | 0.00 | 0.00 | 0.00 | 0.10 | 0.00 | 0.00 | 0.00 | 0.00 | 0.00 |
| 2 | 0.00 | 0.00 | 0.00 | 0.05 | 0.00 | 0.02 | 0.00 | 0.00 | 0.84 | 0.00 | 0.00 | 0.01 | 0.00 | 0.00 | 0.05 | 0.00 | 0.00 | 0.00 | 0.00 | 0.00 |
| 3 | 0.00 | 0.00 | 0.00 | 0.07 | 0.00 | 0.04 | 0.00 | 0.00 | 0.88 | 0.00 | 0.00 | 0.00 | 0.00 | 0.00 | 0.00 | 0.00 | 0.00 | 0.00 | 0.00 | 0.00 |
| 4 | 0.00 | 0.00 | 0.00 | 0.00 | 0.00 | 0.85 | 0.00 | 0.00 | 0.00 | 0.00 | 0.00 | 0.00 | 0.00 | 0.00 | 0.00 | 0.11 | 0.02 | 0.00 | 0.00 | 0.00 |
| 5 | 0.00 | 0.00 | 0.00 | 0.00 | 0.20 | 0.00 | 0.00 | 0.22 | 0.00 | 0.25 | 0.12 | 0.00 | 0.00 | 0.00 | 0.00 | 0.00 | 0.00 | 0.20 | 0.00 | 0.00 |
| 6 | 0.00 | 0.00 | 0.00 | 0.00 | 0.03 | 0.00 | 0.00 | 0.08 | 0.15 | 0.15 | 0.46 | 0.00 | 0.00 | 0.00 | 0.00 | 0.00 | 0.03 | 0.08 | 0.00 | 0.00 |
| 7 | 0.00 | 0.00 | 0.22 | 0.69 | 0.00 | 0.05 | 0.00 | 0.00 | 0.01 | 0.00 | 0.00 | 0.00 | 0.00 | 0.01 | 0.00 | 0.00 | 0.00 | 0.00 | 0.00 | 0.00 |
| 8 | 0.00 | 0.00 | 0.00 | 0.00 | 0.00 | 0.00 | 0.00 | 0.00 | 0.89 | 0.00 | 0.01 | 0.04 | 0.00 | 0.03 | 0.00 | 0.00 | 0.00 | 0.00 | 0.00 | 0.00 |
| 9 | 0.00 | 0.00 | 0.00 | 0.00 | 0.00 | 0.00 | 0.00 | 0.92 | 0.00 | 0.04 | 0.01 | 0.00 | 0.00 | 0.00 | 0.00 | 0.00 | 0.00 | 0.03 | 0.00 | 0.00 |
| 10 | 0.00 | 0.00 | 0.00 | 0.01 | 0.00 | 0.00 | 0.00 | 0.00 | 0.87 | 0.00 | 0.05 | 0.00 | 0.00 | 0.00 | 0.00 | 0.03 | 0.01 | 0.00 | 0.00 | 0.00 |
| 11 | 0.00 | 0.00 | 0.20 | 0.77 | 0.00 | 0.01 | 0.00 | 0.00 | 0.00 | 0.00 | 0.00 | 0.00 | 0.00 | 0.00 | 0.00 | 0.00 | 0.00 | 0.00 | 0.00 | 0.00 |
| 12 | 0.00 | 0.00 | 0.00 | 0.00 | 0.00 | 0.00 | 0.02 | 0.00 | 0.94 | 0.00 | 0.01 | 0.00 | 0.00 | 0.00 | 0.00 | 0.00 | 0.00 | 0.00 | 0.00 | 0.00 |
| 13 | 0.00 | 0.00 | 0.00 | 0.00 | 0.09 | 0.00 | 0.00 | 0.17 | 0.00 | 0.70 | 0.02 | 0.00 | 0.00 | 0.00 | 0.00 | 0.00 | 0.00 | 0.01 | 0.00 | 0.00 |
| 14 | 0.00 | 0.00 | 0.00 | 0.00 | 0.00 | 0.02 | 0.14 | 0.00 | 0.02 | 0.00 | 0.00 | 0.00 | 0.74 | 0.00 | 0.00 | 0.04 | 0.01 | 0.00 | 0.00 | 0.00 |
| 15 | 0.01 | 0.00 | 0.02 | 0.00 | 0.00 | 0.82 | 0.05 | 0.00 | 0.01 | 0.00 | 0.00 | 0.00 | 0.00 | 0.00 | 0.03 | 0.02 | 0.00 | 0.00 | 0.00 | 0.00 |

### Table S2. Amino acid frequencies of the halophytic F-segment.

| | Amino Acid | | | | | | | | | | | | | | | | | | | |
|---|---|---|---|---|---|---|---|---|---|---|---|---|---|---|---|---|---|---|---|---|
| Residue Position | A | C | D | E | F | G | H | I | K | L | M | N | P | Q | R | S | T | V | W | Y |
| 1 | 0.04 | 0.00 | 0.00 | 0.91 | 0.00 | 0.00 | 0.00 | 0.00 | 0.00 | 0.00 | 0.00 | 0.04 | 0.00 | 0.00 | 0.00 | 0.00 | 0.00 | 0.00 | 0.00 | 0.00 |
| 2 | 0.04 | 0.00 | 0.00 | 0.00 | 0.00 | 0.00 | 0.00 | 0.00 | 0.09 | 0.00 | 0.00 | 0.00 | 0.00 | 0.04 | 0.00 | 0.09 | 0.17 | 0.57 | 0.00 | 0.00 |
| 3 | 0.00 | 0.00 | 0.00 | 0.04 | 0.00 | 0.00 | 0.00 | 0.00 | 0.43 | 0.00 | 0.00 | 0.00 | 0.00 | 0.00 | 0.04 | 0.22 | 0.26 | 0.00 | 0.00 | 0.00 |
| 4 | 0.00 | 0.00 | 0.87 | 0.00 | 0.00 | 0.09 | 0.00 | 0.00 | 0.00 | 0.00 | 0.00 | 0.00 | 0.00 | 0.00 | 0.00 | 0.04 | 0.00 | 0.00 | 0.00 | 0.00 |
| 5 | 0.00 | 0.09 | 0.00 | 0.00 | 0.00 | 0.00 | 0.04 | 0.00 | 0.00 | 0.00 | 0.00 | 0.00 | 0.00 | 0.00 | 0.87 | 0.00 | 0.00 | 0.00 | 0.00 | 0.00 |
| 6 | 0.00 | 0.00 | 0.00 | 0.00 | 0.00 | 1.00 | 0.00 | 0.00 | 0.00 | 0.00 | 0.00 | 0.00 | 0.00 | 0.00 | 0.00 | 0.00 | 0.00 | 0.00 | 0.00 | 0.00 |
| 7 | 0.00 | 0.00 | 0.00 | 0.00 | 0.00 | 0.00 | 0.00 | 0.00 | 0.00 | 0.65 | 0.26 | 0.00 | 0.00 | 0.04 | 0.00 | 0.00 | 0.00 | 0.04 | 0.00 | 0.00 |
| 8 | 0.00 | 0.00 | 0.00 | 0.00 | 0.87 | 0.00 | 0.00 | 0.00 | 0.00 | 0.09 | 0.00 | 0.00 | 0.00 | 0.00 | 0.00 | 0.00 | 0.00 | 0.04 | 0.00 | 0.00 |
| 9 | 0.00 | 0.00 | 0.91 | 0.00 | 0.00 | 0.04 | 0.00 | 0.00 | 0.00 | 0.00 | 0.00 | 0.00 | 0.00 | 0.00 | 0.00 | 0.00 | 0.00 | 0.00 | 0.00 | 0.04 |
| 10 | 0.00 | 0.09 | 0.00 | 0.00 | 0.74 | 0.00 | 0.00 | 0.09 | 0.00 | 0.00 | 0.04 | 0.04 | 0.00 | 0.00 | 0.00 | 0.00 | 0.00 | 0.00 | 0.00 | 0.00 |
| 11 | 0.00 | 0.00 | 0.00 | 0.00 | 0.26 | 0.00 | 0.00 | 0.00 | 0.00 | 0.39 | 0.35 | 0.00 | 0.00 | 0.00 | 0.00 | 0.00 | 0.00 | 0.00 | 0.00 | 0.00 |
| 12 | 0.00 | 0.00 | 0.00 | 0.00 | 0.00 | 0.57 | 0.00 | 0.00 | 0.35 | 0.04 | 0.00 | 0.00 | 0.00 | 0.04 | 0.00 | 0.00 | 0.00 | 0.00 | 0.00 | 0.00 |
| 13 | 0.00 | 0.00 | 0.00 | 0.00 | 0.00 | 0.04 | 0.00 | 0.00 | 0.78 | 0.00 | 0.00 | 0.04 | 0.00 | 0.00 | 0.04 | 0.00 | 0.09 | 0.00 | 0.00 | 0.00 |
| 14 | 0.00 | 0.00 | 0.04 | 0.09 | 0.00 | 0.00 | 0.00 | 0.00 | 0.83 | 0.00 | 0.00 | 0.04 | 0.00 | 0.00 | 0.00 | 0.00 | 0.00 | 0.00 | 0.00 | 0.00 |

### Table S3. Amino acid frequencies of the halophytic Y-segment.

| | Amino Acid | | | | | | | | | | | | | | | | | | | |
|---|---|---|---|---|---|---|---|---|---|---|---|---|---|---|---|---|---|---|---|---|
| Residue Position | A | C | D | E | F | G | H | I | K | L | M | N | P | Q | R | S | T | V | W | Y |
| 1 | 0.00 | 0.00 | 0.90 | 0.08 | 0.00 | 0.00 | 0.00 | 0.00 | 0.00 | 0.00 | 0.00 | 0.02 | 0.00 | 0.00 | 0.00 | 0.00 | 0.00 | 0.00 | 0.00 | 0.00 |
| 2 | 0.06 | 0.00 | 0.02 | 0.80 | 0.00 | 0.00 | 0.00 | 0.00 | 0.02 | 0.00 | 0.00 | 0.00 | 0.00 | 0.10 | 0.00 | 0.00 | 0.00 | 0.00 | 0.00 | 0.00 |
| 3 | 0.02 | 0.00 | 0.00 | 0.00 | 0.08 | 0.00 | 0.08 | 0.00 | 0.02 | 0.04 | 0.00 | 0.00 | 0.00 | 0.00 | 0.04 | 0.00 | 0.00 | 0.00 | 0.00 | 0.71 |
| 4 | 0.00 | 0.00 | 0.00 | 0.00 | 0.00 | 0.98 | 0.00 | 0.00 | 0.00 | 0.00 | 0.00 | 0.00 | 0.00 | 0.02 | 0.00 | 0.00 | 0.00 | 0.00 | 0.00 | 0.00 |
| 5 | 0.00 | 0.00 | 0.02 | 0.00 | 0.00 | 0.00 | 0.00 | 0.00 | 0.04 | 0.00 | 0.00 | 0.76 | 0.00 | 0.08 | 0.08 | 0.00 | 0.00 | 0.02 | 0.00 | 0.00 |
| 6 | 0.02 | 0.00 | 0.00 | 0.00 | 0.00 | 0.00 | 0.02 | 0.00 | 0.00 | 0.00 | 0.00 | 0.00 | 0.94 | 0.00 | 0.02 | 0.00 | 0.00 | 0.00 | 0.00 | 0.00 |
| 7 | 0.00 | 0.00 | 0.00 | 0.00 | 0.02 | 0.00 | 0.00 | 0.24 | 0.00 | 0.08 | 0.06 | 0.00 | 0.00 | 0.00 | 0.00 | 0.00 | 0.02 | 0.57 | 0.00 | 0.00 |

### Table S4. Amino acid frequencies of the halophytic S-segment.

| | Amino Acid | | | | | | | | | | | | | | | | | | | |
|---|---|---|---|---|---|---|---|---|---|---|---|---|---|---|---|---|---|---|---|---|
| Residue Position | A | C | D | E | F | G | H | I | K | L | M | N | P | Q | R | S | T | V | W | Y |
| 1 | 0.08 | 0.12 | 0.00 | 0.02 | 0.03 | 0.06 | 0.03 | 0.07 | 0.01 | 0.53 | 0.00 | 0.00 | 0.00 | 0.00 | 0.00 | 0.03 | 0.00 | 0.01 | 0.00 | 0.00 |
| 2 | 0.00 | 0.00 | 0.11 | 0.00 | 0.00 | 0.08 | 0.54 | 0.00 | 0.03 | 0.01 | 0.00 | 0.01 | 0.01 | 0.04 | 0.08 | 0.07 | 0.00 | 0.00 | 0.00 | 0.01 |
| 3 | 0.02 | 0.01 | 0.01 | 0.01 | 0.00 | 0.09 | 0.06 | 0.01 | 0.02 | 0.02 | 0.00 | 0.04 | 0.01 | 0.00 | 0.52 | 0.17 | 0.00 | 0.00 | 0.00 | 0.00 |
| 4 | 0.01 | 0.01 | 0.04 | 0.00 | 0.00 | 0.01 | 0.06 | 0.00 | 0.00 | 0.00 | 0.00 | 0.02 | 0.01 | 0.00 | 0.00 | 0.82 | 0.01 | 0.00 | 0.00 | 0.00 |
| 5 | 0.11 | 0.00 | 0.17 | 0.01 | 0.00 | 0.30 | 0.06 | 0.00 | 0.01 | 0.01 | 0.00 | 0.09 | 0.02 | 0.00 | 0.01 | 0.20 | 0.00 | 0.00 | 0.00 | 0.00 |
| 6 | 0.01 | 0.00 | 0.07 | 0.00 | 0.00 | 0.01 | 0.00 | 0.00 | 0.01 | 0.00 | 0.00 | 0.00 | 0.00 | 0.00 | 0.00 | 0.90 | 0.00 | 0.00 | 0.00 | 0.00 |
| 7 | 0.07 | 0.01 | 0.08 | 0.00 | 0.00 | 0.06 | 0.00 | 0.00 | 0.00 | 0.00 | 0.00 | 0.02 | 0.00 | 0.00 | 0.00 | 0.74 | 0.02 | 0.00 | 0.00 | 0.00 |
| 8 | 0.00 | 0.01 | 0.00 | 0.00 | 0.00 | 0.04 | 0.00 | 0.01 | 0.00 | 0.00 | 0.00 | 0.04 | 0.00 | 0.00 | 0.00 | 0.88 | 0.01 | 0.00 | 0.00 | 0.00 |
| 9 | 0.08 | 0.00 | 0.08 | 0.00 | 0.00 | 0.02 | 0.00 | 0.00 | 0.02 | 0.00 | 0.00 | 0.03 | 0.00 | 0.00 | 0.00 | 0.76 | 0.00 | 0.00 | 0.00 | 0.00 |
| 10 | 0.00 | 0.00 | 0.03 | 0.01 | 0.00 | 0.03 | 0.00 | 0.00 | 0.00 | 0.00 | 0.00 | 0.00 | 0.01 | 0.00 | 0.00 | 0.91 | 0.00 | 0.00 | 0.00 | 0.00 |
| 11 | 0.09 | 0.00 | 0.09 | 0.02 | 0.00 | 0.11 | 0.00 | 0.01 | 0.00 | 0.00 | 0.00 | 0.00 | 0.00 | 0.00 | 0.00 | 0.65 | 0.02 | 0.00 | 0.00 | 0.00 |
| 12 | 0.00 | 0.01 | 0.10 | 0.06 | 0.00 | 0.02 | 0.00 | 0.00 | 0.01 | 0.00 | 0.00 | 0.00 | 0.00 | 0.00 | 0.00 | 0.79 | 0.00 | 0.01 | 0.00 | 0.00 |
| 13 | 0.00 | 0.00 | 0.36 | 0.42 | 0.00 | 0.03 | 0.01 | 0.00 | 0.01 | 0.00 | 0.00 | 0.00 | 0.02 | 0.00 | 0.01 | 0.12 | 0.00 | 0.01 | 0.00 | 0.00 |
| 14 | 0.00 | 0.00 | 0.58 | 0.19 | 0.00 | 0.02 | 0.00 | 0.00 | 0.04 | 0.01 | 0.00 | 0.01 | 0.00 | 0.00 | 0.00 | 0.11 | 0.00 | 0.02 | 0.00 | 0.00 |
| 15 | 0.00 | 0.02 | 0.57 | 0.26 | 0.00 | 0.06 | 0.01 | 0.00 | 0.02 | 0.01 | 0.00 | 0.00 | 0.00 | 0.01 | 0.00 | 0.03 | 0.00 | 0.00 | 0.00 | 0.00 |
